# Comparative study on long-term stability in mandibular sagittal split ramus osteotomy: hydroxyapatite/poly-l-lactide mesh versus titanium miniplate

**DOI:** 10.1186/s40902-019-0192-6

**Published:** 2019-03-01

**Authors:** Young-Wook Park, Hyun-Sik Kang, Jang-Ha Lee

**Affiliations:** Department of Oral and Maxillofacial Surgery, College of Dentistry, Gangneung-Wonju National University, 7 Jukheon-Gil, Gangneung, Gangwondo 25457 South Korea

**Keywords:** Mandibular SSRO, HA/PLLA mesh, Titanium miniplate, Long-term skeletal stability

## Abstract

**Background:**

Resorbable devices have recently been adopted in the field of orthognathic surgery with controversies about their postoperative skeletal stability. Hence, we determined the long-term skeletal stability of unsintered hydroxyapatite/poly-l-lactic acid (HA/PLLA) mesh for osteofixation of mandibular sagittal split ramus osteotomy (SSRO), and compared it with that of titanium miniplate.

**Methods:**

Patients were divided into resorbable mesh and titanium miniplate fixation groups. A comparative study of the change in the mandibular position was performed with preoperative, 1-day, 6-month, and 2-year postoperative lateral cephalograms.

**Results:**

At postoperative 6 months—compared with postoperative 1 day, point B (supra-mentale) was significantly displaced anteriorly in the titanium-fixation group. Moreover, at postoperative 2 years—compared with postoperative 6 months, point B was significantly displaced inferiorly in the titanium-fixation. However, the HA/PLLA mesh-fixation group did not show any significant change with respect to point B postoperatively.

**Conclusions:**

The HA/PLLA mesh-fixation group demonstrated superior long-term skeletal stability with respect to the position of mandible, when compared with the titanium-fixation group.

## Background

Osteofixation stability is one of the prerequisites for successful orthognathic surgery. Recently, the number of complicated surgeries requiring greater magnitude of segmental movements or segmental movements to the position of tissue resistance has increased for maxillofacial plastic and reconstructive surgeons. In particular, mandibular setback remains a more unstable movement than mandibular advancement [1]. Therefore, techniques of modern orthognathic surgery require greater osteofixation stability.

For a long time, titanium plates and screws have been considered as the “gold standard” for rigid fixation in orthognathic surgery. Recently, general consensus has changed from routine removal of the titanium devices to leaving them in the body unless it causes any problems [2]. Although titanium binds to the bone, titanium plates and screws should be removed due to adverse effects to surrounding tissues [3], interference with radiological evaluation, and possible stress-shielding, as well as patient’s request.

We can eliminate the potential need for a second operation by using resorbable osteosynthesis. To the best of our knowledge, the use of resorbable plates in the field of orthognathic surgery was first reported in 1998 [4]. To date, a lot of clinical studies have reported comparable results between the resorbable and titanium devices regarding postoperative skeletal stability and frequency of relapse [5–10]. Some surgeons pointed out the lack of segmental stability, especially in the early postoperative period of resorbable osteosynthesis [11].

However, recent innovative technology produced more rigid resorbable materials, such as unsintered hydroxyapatite/poly-l-lactic acid (HA/PLLA) [12]. In this study, we determined the long-term skeletal stability of HA/PLLA mesh for osteofixation of mandibular SSRO, and compared it with that of titanium miniplate.

## Methods

This study was approved by the institutional review board of Gangneung-Wonju National University Dental Hospital (IRB No. 2017-003). Orthognathic surgeries were performed by one surgeon between 2010 and 2014. The inclusion criteria for patients were as follows:

1. Diagnosis of developmental dentofacial deformity without any congenital anomaly.
2. Mandibular prognathism with or without facial asymmetry.
3. History of mandibular or maxillomandibular surgery using mandibular bilateral SSRO (Obwegesser-Dal Pont technique).
4. History of concomitant advancement or straightening genioplasty.
5. Application of the same osteofixation system to the maxillary and mandibular osteofixation in case of maxillomandibular surgery.

**Fig. 1:**
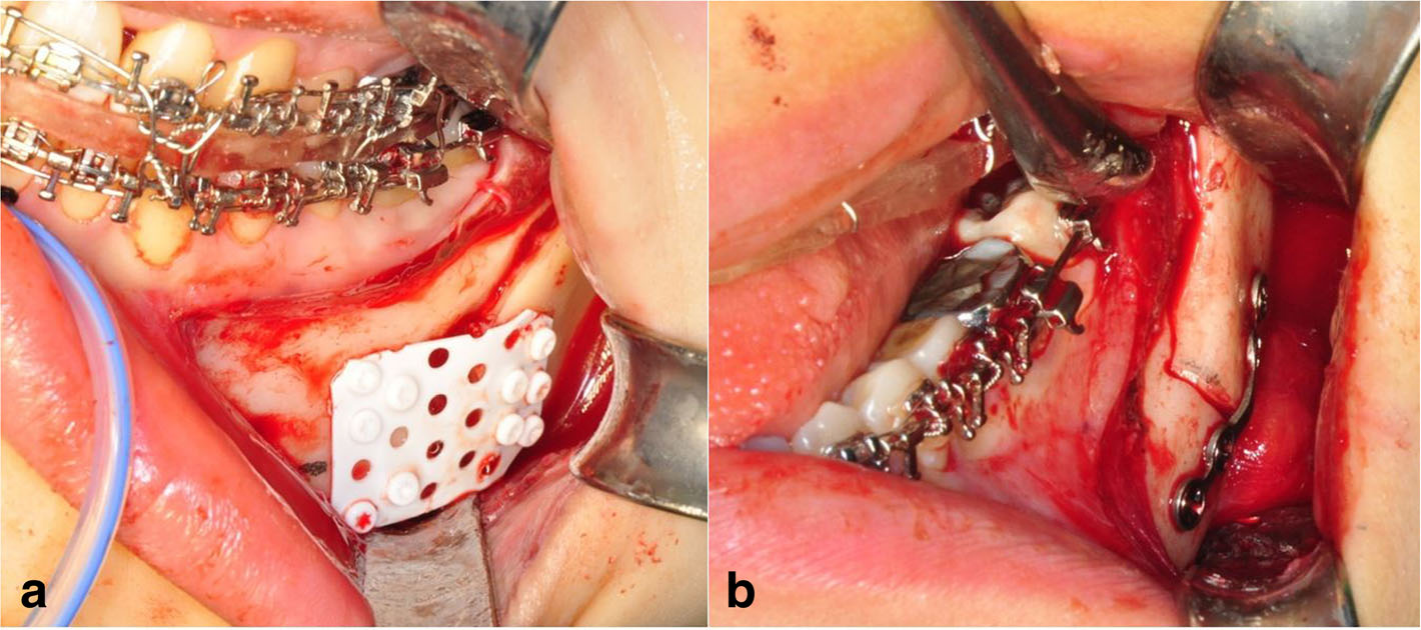
Clinical situations of resorbable HA/PLLA mesh (**a**) and titanium miniplate (**b**) osteofixation in SSRO

**Fig. 2:**
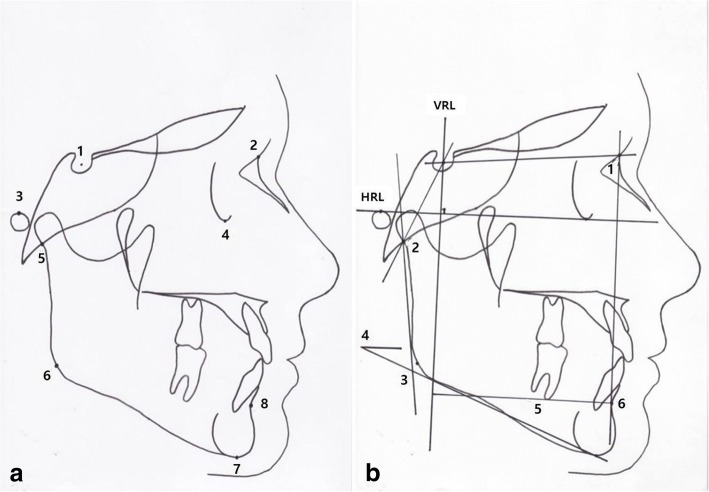
**a** Reference points used in this study: 1. Sella (S); 2. Nasion (N); 3. Porion (Po); 4. Orbitale (Or); 5. Articulare (Ar); 6. Gonion (Go); 7. Menton (Me); 8. Supramentale (B). **b** Measuring parameters used in this study: 1. SNB (°); 2. Articular angle (°); 3. Gonial angle (°); 4. Mandibular plane angle to FH (°); 5. VRL-B (mm); 6. HRL-B (mm)

Patients were divided into 0.5-mm, resorbable mesh (OsteotransMX®, Takiron, Osaka, Japan; group 1) and 2.0-mm, 4-hole titanium miniplate (M3®, Osteomed Co., Addison, TX, USA; group 2) fixation groups. For resorbable-osteofixation of mandibular SSRO, 3–5 resorbable screws with 5.0-mm length were engaged in each segment. Then, 0.5-mm unsintered-HA/PLLA mesh was easily bendable in room temperature to fit the contour of mandibular SSRO site. For titanium-osteofixation, 6.0-mm titanium screws were used (Fig. 1). In the case of maxillomandibular surgery, all patients had undergone surgical alterations of maxilla with unilateral impaction and/or posterior impaction. A comparative study of the change in the mandibular position was performed with preoperative (T1), 1-day (T2), 6-month (T3), and 2-year (T4) postoperative lateral cephalograms using a photoanalysis software Xelis dental® (Infinity care, Seoul, Korea). We set up eight reference points (Fig. 2a) and six measuring parameters (Fig. 2b) in consecutive lateral cephalograms. We used FH plane (porion; Po-orbitale; Or) as the horizontal reference line (HRL), and the vertical reference line (VRL) was defined as the line perpendicular to FH plane over Sella (S) point.

All statistical analyses were done by using IBM SPSS statistics version 23 (IBM Co., NY, USA). The differences between T1 and T2 (T2-T1) determined the surgical change, the differences between T2 and T3 (T3-T2) determined the short-term relapse, and the differences between T3 and T4 (T4-T3) determined the long-term relapse after operation. The Wilcoxon rank sum test was applied to analyze the surgical change, as well as short-term and long-term relapses. Moreover, the Mann-Whitney test was applied to analyze the differences between the groups at each time point. Values of *p* < 0.05 were considered significant.

## Results

A total of 30 patients (16 males and fourteen females) were enrolled in this study. Patients’ basic information is summarized in Table 1. Clinically, no patient experienced operative complications.

**Table 1:** Patient’s information

	Resorbable mesh group (*n* = 16)	Titanium miniplate group (*n* = 14)
Age (years)	22.8 ± 3.1	22.9 ± 5.1
Gender		
Male	7	9
Female	9	5
Number of patients		
Mandible (genioplasty)	5 (2)	7 (5)
Maxillomandible (genioplasty)	11 (5)	7 (2)

### Preoperative stage

On the presurgical stage, no statistical differences were checked in each parameter between the two groups (Table 2). Therefore, there were no significant differences with respect to the skeletal characteristics between the two groups.

**Table 2:** Comparison of absorbable group and titanium group on the presurgical stage

Parameters	Absorbable group (*n* = 16)		Titanium group (*n* = 14)		*P* value
	Mean	SD	Mean	SD	
Horizontal measurements					
VRP-B (mm)	56.83	8.17	52.10	7.96	0.759
Vertical measurements					
HRP-B (mm)	80.80	7.03	79.16	8.75	0.697
Angular measurements					
SNB	82.26	4.90	78.66	3.71	0.637
Articular angle	138.05	7.59	136.04	7.17	0.313
Gonial angle	128.94	8.12	127.35	5.07	0.822
Mandibular plane angle	34.27	6.88	33.28	6.70	0.667

*P* = 0.05

Mann-Whitney test

### Surgical change (T2-T1)

After the operation, point B was moved posterosuperiorly according to the surgical change of the mandibular setback and maxillary impaction. SNB was also significantly decreased in both groups. Otherwise, articular angle, gonial angle, and mandibular plane angle revealed no significant differences in both groups (Table 3).

**Table 3:** Comparison of surgical change (T2-T1) on absorbable group and titanium group

Parameters	Absorbable group (*n* = 16)		Titanium group (*n* = 14)		*P* value
	Mean	*P* value	Mean	*P* value	
Horizontal measurements					
VRP-B (mm)	− 7.39 ± 4.53	0.000†	− 7.18 ± 4.85	0.001†	0.854
Vertical measurements					
HRP-B (mm)	− 2.35 ± 4.07	0.044†	− 2.35 ± 4.29	0.055	0.951
Angular measurements					
SNB	− 3.74 ± 1.73	0.000†	− 4.69 ± 3.12	0.002†	0.355
Articular angle	2.97 ± 6.08	0.098	0.79 ± 5.57	0.660	0.377
Gonial angle	− 0.57 ± 6.47	0.453	− 1.78 ± 5.30	0.198	0.697
Mandibular plane angle	1.13 ± 3.17	0.179	0.59 ± 2.40	0.140	0.580

Wilcoxon rank sum test

Mann-Whitney test

*P* = 0.05

† = Significant difference

### Short-term relapse (T3-T2)

At postoperative 6 months, point B moved to a horizontally anterior position in both groups. However, statistical significance was detected only in group 2 (titanium-fixation group). SNB was also significantly increased in group 2, which indicated short-term relapse. Otherwise, the gonial angle was significantly increased in both groups, and the mandibular plane angle was significantly increased in group 1 (Table 4).

**Table 4:** Comparison of post-surgical change (T3-T2) on absorbable group and titanium group

Parameters	Absorbable group (*n* = 16)		Titanium group (*n* = 14)		*P* value
	Mean	*P* value	Mean	*P* value	
Horizontal measurements					
VRP-B (mm)	1.94 ± 3.61	0.063	2.86 ± 3.55	0.017†	0.580
Vertical measurements					
HRP-B (mm)	− 0.95 ± 2.63	0.173	− 0.87 ± 3.10	0.397	0.984
Angular measurements					
SNB	0.70 ± 1.25	0.056	1.61 ± 1.41	0.001†	0.154
Articular angle	− 2.15 ± 3.94	0.215	− 0.28 ± 4.45	0.975	0.166
Gonial angle	4.77 ± 5.20	0.006†	2.53 ± 4.35	0.048†	0.423
Mandibular plane angle	1.94 ± 3.43	0.039†	0.65 ± 2.78	0.363	0.423

Wilcoxon rank sum test

Mann-Whitney test

*P* = 0.05

† = Significant difference

### Long-term relapse (T4-T3)

At postoperative 2 years, point B moved to a vertically inferior position in group 2. Other parameters did not show any significant differences between the two groups, and compared with postoperative 6 months (Table 5).

**Table 5:** Comparison of long-term result change (T4-T3) on absorbable group and titanium group

Parameters	Absorbable group (*n* = 16)		Titanium group (*n* = 14)		*P* value
	Mean	*P* value	Mean	*P* value	
Horizontal measurements					
VRP-B (mm)	0.28 ± 2.44	0.605	− 0.65 ± 3.81	0.975	0.728
Vertical measurements					
HRP-B (mm)	0.33 ± 2.10	1.000	1.38 ± 2.36	0.048†	0.142
Angular measurements					
SNB	0.20 ± 0.80	0.816	0.00 ± 0.64	0.683	1.000
Articular angle	− 1.14 ± 3.73	0.148	− 0.22 ± 2.90	0.754	0.790
Gonial angle	0.25 ± 2.28	0.365	− 0.19 ± 2.07	0.778	0.423
Mandibular plane angle	0.58 ± 1.22	0.121	− 0.30 ± 1.74	0.397	0.064

Wilcoxon rank sum test

Mann-Whitney test

*P* = 0.05

† = Significant difference.

## Discussion

Resorbable devices have increasingly been adopted in orthognathic surgery as it eliminates the necessity for patients to undergo a second-stage removal surgery. However, it is worth noting that one of the disadvantages of resorbable devices is the lack of rigidity. However, a lot of clinical studies reported a predictable rigid osteosynthesis with the use of polymers of poly-lactic acid [13–15]. In our long-term follow-up study, we have induced a meaningful data, suggesting that HA/PLLA mesh-osteofixation may be superior to titanium miniplate-osteofixation at the mandibular SSRO site. In our previous animal experiment, we reproduced the clinical situations of HA/PLLA mesh and titanium miniplate-osteofixation after creating a discontinuity defect on rabbit mandible. Through it, we confirmed similar results with this clinical study in terms of recovery of functional rehabilitation evaluated by daily feed intake amount, and incidences of malocclusion and screw loosening [16].

In this study, a total of 30 patients were included. Their skeletal characteristics did not show any significant differences between the experimental (resorbable mesh) and control (titanium miniplate) groups. Eleven out of 16 patients in the experimental group and 7 out of 14 patients in the control group underwent maxillary posterior impaction and mandibular setback. The osteotomy line for genioplasty was located under the supramentale (point B). Therefore, point B was moved posteriorly and superiorly after surgery, regardless of accompanying genioplasty. Therefore, the significant change of point B in the titanium miniplate group indicates relapse tendency. Moreover, the significant changes of gonial angle and mandibular plane angle at postoperative 6 months seem to indicate complex results of segmental remodeling, adaptive change of temporomandibular joints, and postoperative orthodontics.

HA/PLLA mesh, which was used in this study, is a nanocomposite consisting of unsintered hydroxyapatite and carbonate ion combined with poly**-**l**-**lactide. A study reported unsintered-HA/PLLA nanocomposite maintains a bending strength equal to that of human cortical bone for 25 weeks in vivo [17]. Furthermore, the bending strength was maintained at 52 weeks in the subcutis of the rabbit [18]. Therefore, it seems to provide a stable segmental stability, similar to titanium miniplate osteosynthesis [19]. Furthermore, in this study, resorbable mesh-osteofixation, which allows greater screw installation, demonstrated increased reliable horizontal stability than titanium miniplate-osteofixation at postoperative 6 months. This is probably because three to five screws were engaged at each segment after SSRO for resorbable mesh-osteofixation.

For our patients, titanium miniplates were removed about 6 months after the operation. However, the resorbable mesh is supposed to be biodegraded with body fluids and display osteoconduction due to the well-known properties of HA. The biodegradation period was reported to be 4.5–5.5 years [20]. At postoperative 2 years, the mechanical strength of HA/PLLA mesh was almost lost, but still—to some extent—maintained. This is why HA/PLLA mesh-osteofixation demonstrated more reliable vertical stability than titanium miniplate-osteofixation at postoperative 2 years. During the degradation period, a localized inflammation had developed in one patient in group 1, which was controlled unevenfully by surgical debridement and antibiotics. Rarely, metabolic products of poly-lactic acid may trigger a foreign-body inflammatory reaction [21].

Another advantage of HA/PLLA mesh is its compact design. From the authors’ experience with polymers of PLLA, larger devices, such as a 2.4 mm, 6-hole plate, should be used to achieve a stability at the SSRO site [22]. It may sometimes be palpable after operation when the patient has thin skin. However, in this clinical study, we have adopted a 0.5-mm-thick mesh that is easily cut and adaptable at the SSRO site. The limitations of our study is that the number of samples is very small and various surgical methods which likely to affect on the results were performed.

## Conclusions

Titanium miniplate-osteofixation of the SSRO site revealed short-term horizontal and long-term vertical relapse in mandibular position in patients with mandibular prolaterognathism. However, HA/PLLA mesh-osteofixation of SSRO site demonstrated stable mandibular position postoperatively. Therefore, we can induce a greater reliable skeletal stability by using mesh-type resorbable device. This may be the case since resorbable mesh has spatial advantage in terms of screw installation, which could be confirmed by a finite element method.

## Abbreviation

n: Number
